# An abundance of Epsilonproteobacteria revealed in the gut microbiome of the laboratory cultured sea urchin, *Lytechinus variegatus*

**DOI:** 10.3389/fmicb.2015.01047

**Published:** 2015-10-13

**Authors:** Joseph A. Hakim, Hyunmin Koo, Lacey N. Dennis, Ranjit Kumar, Travis Ptacek, Casey D. Morrow, Elliot J. Lefkowitz, Mickie L. Powell, Asim K. Bej, Stephen A. Watts

**Affiliations:** ^1^Department of Biology, University of Alabama at BirminghamBirmingham, AL, USA; ^2^Biomedical Informatics, Center for Clinical and Translational Sciences, University of Alabama at BirminghamBirmingham, AL, USA; ^3^Department of Microbiology, University of Alabama at BirminghamBirmingham, AL, USA; ^4^Cell, Developmental, and Integrative Biology, University of Alabama at BirminghamBirmingham, AL, USA

**Keywords:** egested fecal pellet, illumina MiSeq, 16S rRNA, QIIME, Gulf of Mexico, microbiome

## Abstract

In this study, we have examined the bacterial community composition of the laboratory cultured sea urchin *Lytechinus variegatus* gut microbiome and its culture environment using NextGen amplicon sequencing of the V4 segment of the 16S rRNA gene, and downstream bioinformatics tools. Overall, the gut and tank water was dominated by Proteobacteria, whereas the feed consisted of a co-occurrence of Proteobacteria and Firmicutes at a high abundance. The gut tissue represented Epsilonproteobacteria as dominant, with order Campylobacterales at the highest relative abundance (>95%). However, the pharynx tissue was dominated by class Alphaproteobacteria. The gut digesta and egested fecal pellets had a high abundance of class Gammaproteobacteria, from which *Vibrio* was found to be the primary genus, and Epsilonproteobacteria, with genus *Arcobacter* occurring at a moderate level. At the class level, the tank water was dominated by Gammaproteobacteria, and the feed by Alphaproteobacteria. Multi-Dimensional Scaling analysis showed that the microbial community of the gut tissue clustered together, as did the pharynx tissue to the feed. The gut digesta and egested fecal pellets showed a similarity relationship to the tank water. Further analysis of Campylobacterales at a lower taxonomic level using the oligotyping method revealed 37 unique types across the 10 samples, where Oligotype 1 was primarily represented in the gut tissue. BLAST analysis identified Oligotype 1 to be *Arcobacter* sp., *Sulfuricurvum* sp., and *Arcobacter bivalviorum* at an identity level >90%. This study showed that although distinct microbial communities are evident across multiple components of the sea urchin gut ecosystem, there is a noticeable correlation between the overall microbial communities of the gut with the sea urchin *L. variegatus* culture environment.

## Introduction

Recent advancements in the discovery of gut microbial communities in the animal kingdom has offered a glimpse into the supportive role of various microbial taxa in growth, development, metabolism, and digestive physiology of the host, as well as protection from predators, and adaptive fitness to the environment they inhabit (Shin et al., 2011; Gomez et al., 2012; Nguyen and Clarke, 2012; Guinane and Cotter, 2013; Kostic et al., 2013; Heintz and Mair, 2014). Conventional microbiological culture-based methods, and more recently the advent of the culture-independent NextGen sequencing approach, has enhanced our capability to understand the gut microbial composition of many animals with the highest coverage, and in particular, a number of invertebrates such as Crustacea, Mollusca, and some Echinodermata (Harris, 1993; King et al., 2012; Gerdts et al., 2013; Kostic et al., 2013; Chauhan et al., 2014). Besides determining the microbial community profile of these invertebrates, the predictive roles of various microbial taxa in both the digestive health of the host, as well as the ecological importance of those bacteria to the host's community has been proposed. Among many ecologically and commercially important invertebrates, the sea urchin has received attention for its importance in the seafood industry (Muraoka, 1990; Andrew et al., 2002), as a model organism for developmental biology (McClay, 2011), and its role in nutrient cycling effecting the community structure and dynamics in the ecosystem they inhabit (Sauchyn and Scheibling, 2009a,b; Sauchyn et al., 2011). Yet, relatively little attention has been given to the sea urchin gut microbial ecology, and the potential role of those microbes in host health and other facets of its natural community (Becker et al., 2007, 2008, 2009; Lawrence et al., 2013).

Lasker and Giese (1954) first proposed a role of microbiota in nutrient digestion and absorption in sea urchins (Lasker and Giese, 1954), and in fact, most of the previous microbial analysis work on the sea urchin has focused on a generalized role of microbes in digestive support (Lawrence et al., 2013), or in disease progression (Becker et al., 2007, 2008, 2009). Later examinations would suggest involvement of the sea urchin gut egesta bacteria in nutrient transfer among trophic levels in their communities (Sauchyn and Scheibling, 2009a,b). Nevertheless, as the microbial ecosystems of the sea urchin gut continue to foretell a relationship between the microbial community and nutrient intake, determining the bacterial composition within the gut of the sea urchin fed a formulated diet in an aquaculture environment would provide valuable insights into sea urchin digestive physiology and health.

The variegated sea urchin, *Lytechinus variegatus* is often found in nearshore seagrass communities in the Gulf of Mexico, and consumes a wide variety of plant and animal material (Watts et al., 2013). In the laboratory culture environment, *L. variegatus* can process formulated diets containing macronutrients from a variety of sources (Hammer et al., 2012). Since gut microbiota has previously been implicated in the digestive process of sea urchins (Lasker and Giese, 1954; Fong and Mann, 1980; Sawabe et al., 1995), understanding the microbial composition of the sea urchin digestive system may elucidate the role of the gut microbiome in conferring host health through formulated diet. In this study, we describe the microbiome composition in the lumen of the digestive tract and gut digesta, along with egested fecal pellets, feeds, and the culture environment with high taxonomic coverage using a culture-independent method of NextGen sequencing technology and bioinformatics tools. The results from this study will help establish the microbial population that is conferred onto the sea urchin through the aquaculture conditions, as well as the trends of distribution and selective enrichment of the microbial community associated with the sea urchin, *L. variegatus*.

## Materials and methods

### Collection and culture of *L. variegatus*

Adult sea urchins were collected on April 2013, from Port Saint Joseph, Florida (29.80° N 85.36° W), and transported in seawater to a recirculating salt water system within the laboratory at the University of Alabama at Birmingham. Water conditions were maintained at 22 ± 2°C, with a pH of 8.2 ± 0.2 and a salinity of 32 ± 1 ppt. using synthetic sea salt (Instant Ocean; Spectrum Brands, Inc., Blacksburg, VA) added to treated municipal water. Prior to use, municipal water was filtered by 5 micron sediment, charcoal, and reverse osmosis membranes, followed by an ion exchange resin, with the final addition of Instant Ocean sea salts to achieve the desired salinity of 32 ppt. Water was replaced in the recirculating seawater culture system at a rate of ca. 5% water exchange per day. Water quality was maintained using a dolomite mechanical gravel filter, followed by biological filtration using Bioballs biological media (Foster and Smith, Inc., Rhinelander, WI), and UV sterilization of water exiting the recirculating filter. The sea urchins were fed a formulated feed (Hammer et al., 2006) *ad libitum*, consisting of a relative percentage of 6% lipid, 28% protein, and 36% carbohydrate, once every 24–48 h for a 6 month period prior to analysis.

### Sample and DNA preparation

Two laboratory-cultivated sea urchins were used for the study (UR1 *d* = 50 mm, wet wt = 60.3 g, and UR2 *d* = 49 mm, wet wt = 63.2 g during the time described in the previous section). Sample collection from each sea urchin began 22 ± 1 h after feeding. Prior to dissection, the sea urchins were relocated to a temporary container containing sterile (autoclaved at 121°C for 20 min at 103.42 kPa) sea water, from which the egested fecal pellets from each sea urchin were collected. After fecal pellet collection, the sea urchins were then removed from the water and dissected immediately. Briefly, an incision was made with sterilized scissors into the test surrounding the peristomial membrane, and a dissection was performed circumnavigating the mouth. The peristomial membrane, along with the nested mouth (the Aristotle's lantern) (Sodergren et al., 2006), was lifted from the sea urchin, while still maintaining the integrity of the digestive tract (Watts et al., 2013).

The pharynx enclosed by the lantern was separated from the digestive tract, collected intact, and rinsed with autoclaved sea water. The remaining segment of the digestive tract (gut tissue), which included the esophagus, stomach, and intestine (Holland, 2013), was then removed from the sea urchin. The gut was rinsed with autoclaved sea water, and voided of gut food pellets by gentle shaking. The gut tissue was collected separately from the gut food pellets and both were rinsed with autoclaved sea water. The microbiota obtained from the seawater within the closed recirculating system where the sea urchins were maintained was collected via vacuum filtration through Millipore 0.22μm filtration paper (EMD Millipore Corporation, Danvers, MA), and feeds were collected from the stock sea urchin food source (Hammer et al., 2006). All samples were divided into 3 separate sub-samples, flash frozen in liquid nitrogen, and preserved at −80°C until used for DNA purification and preparation for sequencing of the 16S rRNA gene. Food samples and whole filter paper containing water system microbes were also divided into three subsamples, frozen in liquid nitrogen, and preserved at −80°C until used.

### Metacommunity DNA purification and generation of 16S rRNA amplicon library

Microbial community DNA was isolated using the Fecal DNA isolation kit from Zymo Research (Irvine, CA; catalog # D6010) following the manufacturer's instructions. Once the sample DNA was prepared, PCR was used with unique bar coded primers to amplify the hyper variable region 4 (V4) of the 16S rRNA gene, to create an amplicon library from metacommunity DNA samples (Kozich et al., 2013; Kumar et al., 2014). The oligonucleotide primers used for the PCR amplification of the V4 region of the 16S rRNA gene were as follows: Forward primer V4: 5′-AAT GATACGGCGACCACCGAGATCTACACTATGGTAATTGTGTGCCAGCMGCCGCGG TAA-3′; and Reverse primer V4: 5′-CAA GAGAAGACGGCATACGAGATNNNNNNAGTCAGTCAGCCGGACTACHVGGGTWTC TAAT-3′ (Eurofins Genomics, Inc., Huntsville, AL) (Kumar et al., 2014). The individual PCR reactions were set up as follows: 10 μL of 5X Reaction Buffer; 1.5 μL (200 μM) of each of the dNTPs; 2 μL (1.5 μM) of each of the oligonucleotide primers; 1.5 μL (5 U) of the “LongAmp” enzyme kit (New England Biolabs, Ipswich, MA; cat # E5200S); 30 μL (2–5 ng/μl) of the template DNA; and 3 μL of sterile H_2_O to a total reaction volume of 50 μL. The PCR cycling parameters were as follows: initial denature 94°C for 1 min; 32 cycles of amplification in which each cycle consisted of 94°C for 30 s, 50°C for 1 min, 65°C for 1 min; followed by final extension of 65°C for 3 min; then a final hold at 4°C. Following PCR amplification of the targeted gene, the entire PCR reaction was electrophoresed on a 1.0% (w/v) Tris-borate-EDTA/agarose gel. The PCR product (approximately 380 bp predicted product size) was visualized by UV illumination. The amplified DNA band was excised with a sterile scalpel, and purified from the agarose matrix using QIAquick Gel Extraction Kit according to manufacturer's instructions (Qiagen, Inc., Venlo, Limburg; cat # 28704).

### Nextgen sequencing and bioinformatics tools

The PCR products were sequenced using the NextGen sequencing Illumina MiSeq platform (Caporaso et al., 2012; Kozich et al., 2013; Kumar et al., 2014). We used a 250 bp paired-end kit from Illumina for the microbiome analysis. The samples were first quantified using Pico Green dye (Life Technologies, Grand Island, NY), adjusted to a concentration of 4 nM, then used for sequencing on the Illumina MiSeq (Kumar et al., 2014). The raw sequence data was then de-multiplexed and converted to FASTQ format (http://maq.sourceforge.net/fastq.shtml). The FASTQ files were subjected to quality assessment using FASTQC (http://www.bioinformatics.babraham.ac.uk/projects/fastqc/), prior to merging and trimming of the raw sequence data, which was followed by quality filtering using the FASTX toolkit (http://hannonlab.cshl.edu/fastx_toolkit/). Since the overlap between the paired reads from each 16S fragment was approximately 245 bases, the overlapping paired end regions were merged to generate a single high quality read, using the “fastq_mergepairs” module of USEARCH (Edgar, 2010). Read pairs with an overlap of less than 50 bases or with mismatches (>20) in the overlapping region were discarded. The sequences were again checked for quality using FASTQC, which was followed by chimeric filtering using the “identify_chimeric_seqs.py” module of USEARCH (Edgar, 2010). The remainder of the steps were performed with the Quantitative Insights into Microbial Ecology microbiome analysis package (QIIME, v1.7.0) (http://qiime.org/) (Lozupone et al., 2007; Caporaso et al., 2010b; Navas-Molina et al., 2013; Kumar et al., 2014). Sequences were grouped into Operational Taxonomic Units (OTUs) using the clustering program UCLUST at a similarity threshold of 97% (Edgar, 2010). The Ribosomal Database Program (RDP) classifier was used to make taxonomic assignments (to the species level wherever possible) for all OTUs at a confidence threshold of 80% (0.8) (Wang et al., 2007). The RDP classifier (http://rdp.cme.msu.edu/) was trained using the Greengenes (v13.8) 16S rRNA database (http://greengenes.lbl.gov/cgi-bin/nph-index.cgi) (McDonald et al., 2011). The resulting OTU table included all OTUs, their taxonomic identification and abundance information. Additionally, OTUs whose average abundance was less than 0.0005% were filtered out. Remaining OTUs were then grouped together to summarize taxon abundance at different hierarchical levels of taxonomic classification (e.g. phylum, class, order, family, and genus). These taxonomy tables were also used to generate stacked column bar charts of taxon abundance using Microsoft Excel software (Microsoft, Seattle, WA). Multiple sequence alignment of OTUs was performed with PyNAST (Caporaso et al., 2010a). Subsampling was performed using the “single_rarefaction.py” module of QIIME (v1.7.0), to account for variation in read depth across samples, (Gotelli and Colwell, 2011), at an even sampling depth of 77,194 reads per sample. The subsampled OTU table was used for downstream Beta and Alpha diversity analyses. A heatmap with the top 25 most highly abundant (>1% in any sample) taxa at the order level was generated using the “heatmap.2” function in R package (available at http://CRAN.R-project.org/package=gplots). The raw sequence files from this study are deposited in the NCBI SRA (http://www.ncbi.nlm.nih.gov/sra), under the accession number SRP062365.

### Oligotyping of the V4 hypervariable region of the campylobacterales 16S rRNA gene

Oligotyping utilizes informative nucleotide variations between similarly clustered reads to designate an oligotype identity (Eren et al., 2013, 2014; Schmidt et al., 2014). After assignment of taxonomy for the total 1,137,478 quality reads, 296,777 sequences from the 10 samples were aligned using MUSCLE, which was implemented in MEGA software (Tamura et al., 2013). The aligned sequences were then used for oligotyping (Eren et al., 2013). After the initial Shannon entropy analysis, 29 variable sites were identified for oligotyping. The parameters required that each oligotype must (1) appear in at least one sample and (2) have a minimum abundance of 100 sequences for each unique oligotype. After elimination of oligotypes not meeting these parameters, 275,566 reads (92.85%) were retained. Each oligotype representative sequence was aligned to the NCBI non-redundant (nr) database using BLAST (http://blast.ncbi.nlm.nih.gov/Blast.cgi).

### Statistical analyses of bacterial diversity

The alpha diversity (diversity within the samples) of the sea urchin microbiome and the culture environment was determined using QIIME (v1.7.0). The alpha-diversity was estimated using observed OTUs, Shannon diversity index (Shannon, 1948; Hill et al., 2003; Marcon et al., 2014), and Simpson diversity index (Simpson, 1949; Hill et al., 2003). In order to estimate the beta diversity (differences between the samples), the OTUs of the bacterial communities were analyzed using Primer-6 analytical software (Primer-E Ltd., Plymouth Marine Laboratory, Plymouth U.K., v6.1.2) (www.primer-e.com). Discrete OTU counts per sample were standardized, and then transformed to the square root values (Clarke and Gorley, 2001). Multidimensional scale plots (Kruskal and Wish, 1978; Clarke, 1993; Clarke and Gorley, 2001), were generated according to Bray–Curtis similarity values (Bray and Curtis, 1957; Clarke and Gorley, 2001).

## Results

### Total illumina sequence reads, quality trimming, and OTU designation

A total of 1,481,476 raw sequence reads of the V4 segment of the 16S rRNA gene from 10 samples of the two sea urchin (UR1 and UR2) gastrointestinal tracts, feeds, and tank water, were generated on an Illumina Miseq sequencing platform (Table 1). The sea urchin microbiome samples consisted of the gut tissues, pharynx tissues, gut digesta, and egested fecal pellets. After high stringent quality-based trimming, 1,137,478 quality sequence reads were used for further bioinformatics analyses. Within these reads, 181,169 sequences clustered into 609 OTUs from the gut tissue; 221,150 sequences clustered into 2,455 OTUs from the pharynx tissue; 219,512 sequences clustered into 926 OTUs from the egested fecal pellets; 204,048 sequences clustered into 1,562 OTUs from the gut digesta; 164,930 sequences clustered into 1,654 distinct OTUs from the sea urchin feed; and lastly 146,669 reads clustered into 1,511 OTUs from the tank water (Table 1). All OTUs were clustered at a 97% sequence similarity from the trimmed sequences of the respective samples using UCLUST (Edgar, 2010; Koo et al., 2014).

**Table 1:** **Sample statistics following NextGen sequencing and the diversity values, as determined by QIIME (v1.7.0), are listed**.

**Sample**	**Raw Sequences**	**Trimmed Sequences**	**OTUs Identified**	**Shannon**	**Simpson**
Tank water	181,387	146,669	1511	6.51	0.95
Sea urchin feed	205,651	164,930	1654	5.68	0.93
UR1 Pharynx tissue	138,911	97,670	1190	6.21	0.95
UR2 Pharynx tissue	180,891	123,480	1265	6.16	0.96
UR1 Gut tissue	90,693	77,194	188	0.17	0.02
UR2 Gut tissue	127,431	103,975	421	0.56	0.09
UR1 Gut digesta	120,424	100,073	861	3.87	0.76
UR2 Gut digesta	176,771	103,975	701	3.39	0.78
UR1 Egested fecal pellet	128,082	110,922	384	2.79	0.65
UR2 Egested fecal pellet	131,235	108,590	542	3.71	0.81

*Included are the number of raw sequences, trimmed sequences, and unique OTUs. Shannon and Simpson diversity indices are also presented. UR1, sea urchin 1; UR2, sea urchin 2*.

### Microbial diversity across different samples

**Figure 1:**
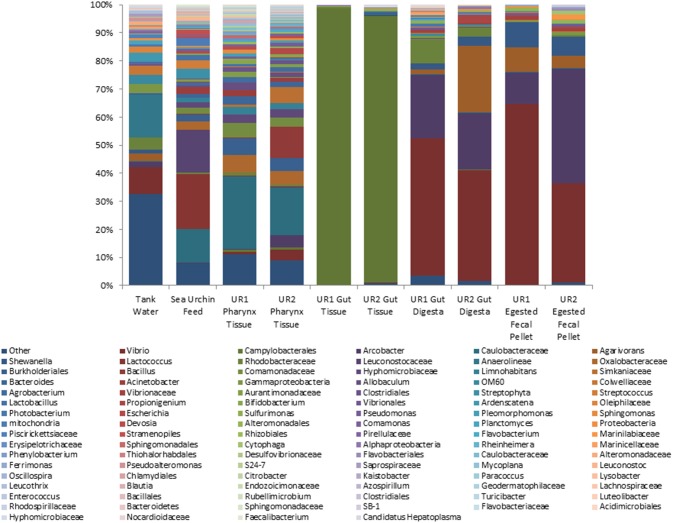
**Stacked column bar graph depicting the relative abundances and distribution of the most highly abundant resolved taxa across the 10 samples of this study**. The gut microbiome consisted mainly of Phylum Proteobacteria, whereas the sea urchin feed was dominated by both Firmicutes and Proteobacteria. At the highest resolution, order Campylobacterales was determined to be the most abundant taxa in the gut tissue. In the gut digesta and egested fecal pellets, *Vibrio, Arcobacter*, and *Agarivorans* were observed. Relative abundances were performed through QIIME (v1.7.0), and graphs were generated using Microsoft Excel software (Microsoft, Seattle, WA). UR1, sea urchin 1; UR2, sea urchin 2.

The relative abundances of taxa identified to the most resolvable taxa (phylum, class, order, family, and genus) across all 10 samples are elaborated in Figure 1. In the gut tissue samples of the sea urchins, microorganisms belonging to phylum Proteobacteria represented the highest relative abundance. Further analysis revealed class Epsilonproteobacteria to be dominant, and from within this class, order Campylobacterales was the most abundant taxon. Resolution to the genus level could not be achieved in the gut tissue samples. The pharynx tissue of the sea urchins was also dominated by Proteobacteria, and at the class level, Alpha-, Beta-, Epsilon-, and Gammaproteobacteria were presented. *Arcobacter, Mycoplana*, and *Vibrio* appeared as the highly represented genera from phylum Proteobacteria. Phylum Firmicutes was represented by a high relative abundance of the genera *Bacillus* and *Allobaculum*.

The gut digesta consisted mainly of bacteria belonging to phylum Proteobacteria, with class Gammaproteobacteria being distinguishably elevated. The dominant genera were *Agarivorans, Arcobacter, Shewanella*, and *Vibrio*, all of which belonging to phylum Proteobacteria. The bacterial composition in the egested fecal pellets consisted of many of the same taxa observed in the gut digesta. In the egested fecal pellets, Proteobacteria accounted for the highest abundance, and at the class level, Gammaproteobacteria was dominant. At the genus level, *Agarivorans, Arcobacter, Shewanella*, and *Vibrio* were detected as dominant taxa.

The microbiota of the sea urchin feed consisted of phylum Proteobacteria, as well as Firmicutes at the highest abundance. Classes Alpha- and Betaproteobacteria were dominant in the feed, and at the genus level, *Agrobacterium, Acinetobacter, Limnohabitans*, and *Mycoplana* were observed. From phylum Firmicutes, order Lactobacillales dominated in the feed, and at the genus level, *Lactobacillus, Lactococcus, Leuconostoc*, and *Streptococcus* were observed. The microbial composition of the tank water was found to be more diverse as compared to the other samples. Of the represented phyla, Proteobacteria was found to be dominant, followed by Chloroflexi, and to a lesser extent Bacteroidetes. Classes Gamma- and Alphaproteobacteria were dominant, and at the order level, Alteromonadales and Vibrionales were represented at relatively high abundances. In addition, significant abundances of genera *Arcobacter, Agarivorans, Shewanella, Pseudoalteromonas*, and *Vibrio* were identified within phylum Proteobacteria. For all samples, the taxonomic groups identified at the genus level have been elaborated in Supplementary Table 1.

### Differentiation of distinct taxa using oligotyping methods and blast

**Figure 2:**
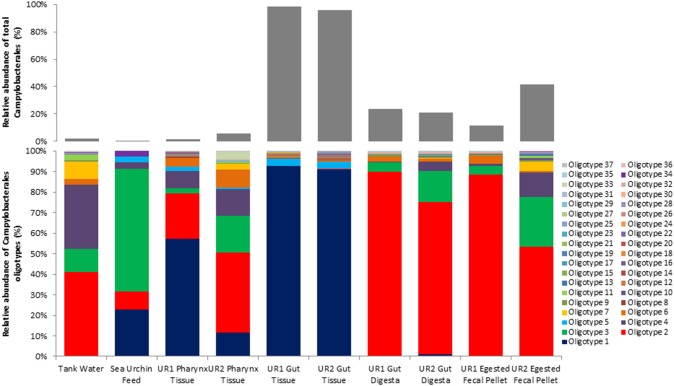
**Oligotype distributions for the 10 samples used in this study**. The relative abundance of each oligotype within the total Campylobacterales diversity for each sample is presented in stacked column bar graphs (bottom), and the proportion of the relative abundance of total Campylobacterales within all bacterial diversity for each sample is shown with light gray bars (top). Oligotyping analyses were performed using the open-source pipeline for oligotyping, available at http://oligotyping.org. The stacked column bar graphs were generated using Microsoft Excel software (Microsoft, Seattle, WA). UR1, sea urchin 1; UR2, sea urchin 2.

Oligotyping analysis of those sequences corresponding to order Campylobacterales in the 10 samples of this study revealed 37 different oligotypes (Figure 2; UR1, sea urchin 1, UR2, sea urchin 2). Of these oligotypes, 21 were found in the UR1 and 11 in the UR2 gut tissues; 21 in the UR1 and 30 in the UR2 pharynx tissues; 17 in the UR1 and 26 in the UR2 gut digesta; 18 in the UR1 and 17 in UR2 egested fecal pellets. The tank water and feed contained 18 and 6 oligotypes, respectively. Of all the identified oligotypes, Oligotype 1 was found to be overrepresented in the gut tissues of the sea urchins, with a relative abundance of 92.7% for UR1 and 91% for UR2. This oligotype was detected in the tank water at 0.3%, and the sea urchin feed at 22.8% (Figure 2). Across all samples, Oligotype 2 (which ranged from 8.5% to 88.36%) and Oligotype 3 (2.3% to 60%) were highly abundant, except for the gut tissues (Figure 2). A MEGABLAST search of the representative sequence of Oligotype 1 displayed a close match to an uncultured *Arcobacter* sp. clone (Identity: 91%, *E*-value: 1.82E–87), *Arcobacter bivalviorum* (Identity: 91%, *E*-value: 2.00e–89), *Sulfuricurvum* sp. (Identity: 90%, *E*-value: 4.00E–86), and an uncultured bacterium clone (Identity: 90%, *E*-value: 2.00e-89; Supplementary Table 2). A MEGABLAST search was performed on the other 36 identified oligotypes, revealing most to be closely related to uncultured *Arcobacter* sp., or uncultured bacterium clones (Supplementary Table 2).

### Statistical analysis

**Figure 3:**
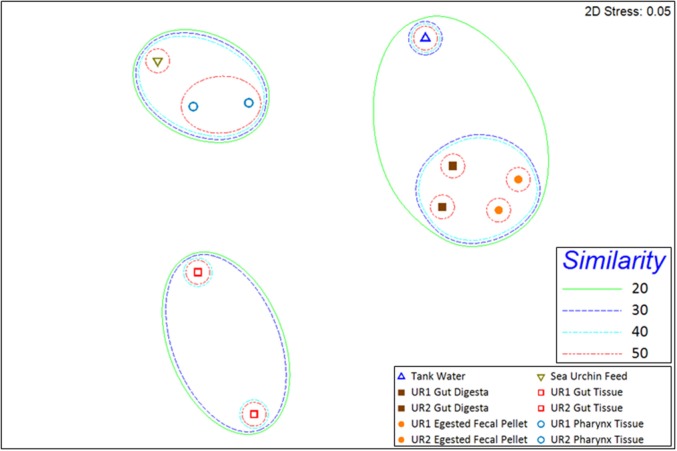
**2D multidimensional scaling (MDS) graph generated through PRIMER-6 (www.primer-e.com)**. Overlay of similarity clusters were produced according to Bray–Curtis Similarity values, set at 10% intervals from 20% to 50%. The pharynx tissue and sea urchin feed sample microbial ecologies clustered with a similarity greater than 40%. The tank water, gut digesta, and egested fecal pellet samples also clustered together at a similarity greater than 20%. The gut tissue samples from the two sea urchins showed a divergent cluster pattern, illustrating a reduced degree of similarity to the other samples of the study. UR1, sea urchin 1; UR2, sea urchin 2. Similarity = Bray–Curtis Similarity (scaled to 100).

Rarefaction curves representing the number of unique OTUs from the normalized 16S rRNA sequences obtained from two sea urchins and their environments (total of 10 samples) reached or approached a plateau, indicating that a sufficient sequencing depth was used to assess community diversity (Supplementary Figure 1). Shannon (Shannon, 1948; Hill et al., 2003; Marcon et al., 2014) and Simpson diversity indices (Simpson, 1949; Hill et al., 2003) displayed relatively low diversity within the gut tissue samples, whereas moderate diversity within egested fecal pellet and gut digesta samples; and high diversity within pharynx tissue, sea urchin feeds, and tank water samples (Table 1). The multidimensional-scaling (MDS) plot (Kruskal and Wish, 1978; Clarke, 1993; Clarke and Gorley, 2001) revealed three distinct clusters of similarity among corresponding samples from the two sea urchins (Figure 3). In the MDS plot, the first dimension of gut tissues were differentiated from all other samples, and the second dimension separated the pharynges and feeds from the rest of the samples, i.e., the egested fecal pellet, gut digesta, and tank water (Figure 3). Subsampling of OTUs showed no significant differences in the cluster patterns of microbial communities in the respective samples.

**Figure 4:**
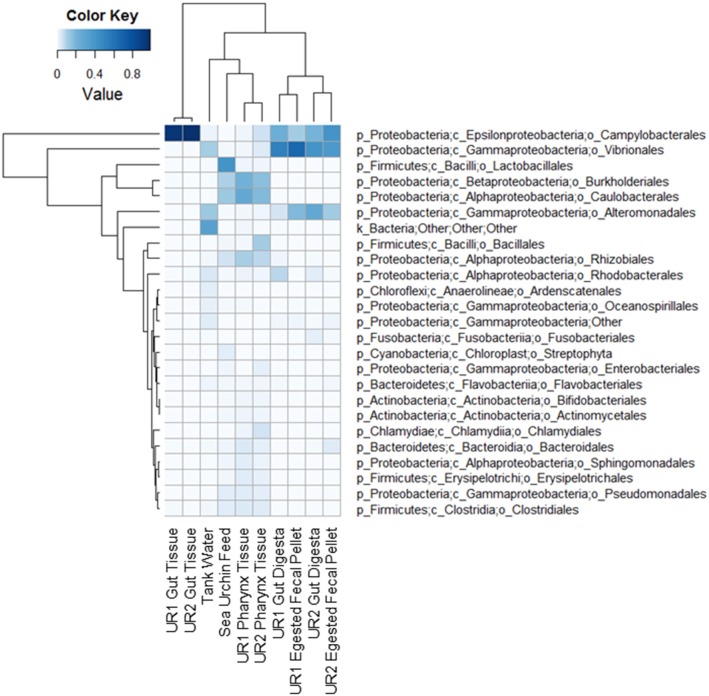
**Heatmap of microbial compositions at the order level**. The rows represent the bacterial taxa and the columns represent the 10 samples used in this study. Both dendrograms were created using hierarchical clustering (complete linkage) of the compositional data. The heatmap was generated using the “heatmap.2” function in R package (available at http://CRAN.R-project.org/package=gplots). UR1, sea urchin 1; UR2, sea urchin 2.

Inter-sample microbial community compositions showed a similarity between samples (Figure 4). The gut tissue revealed a significant abundance of members from order Campylobacterales. The presence of Campylobacterales was also observed to be highly abundant in the gut digesta and egested fecal pellets, along with a significant presence of order Vibrionales. In the pharynx tissue, orders Burkholderiales and Caulobacterales were found to be abundant, whereas the tank water had high representation of order Alteromonadales, and the feed had a significant presence of Lactobacillales. The feed also presented orders Burkholderiales and Caulobacterales (Figure 4).

## Discussion

Our study revealed that, although the sea urchin *L. variegatus* has a primitive gut as compared to the highly compartmentalized digestive systems in higher order deuterostomes (Sauchyn et al., 2011; Holland, 2013), distinct microbial compositions and abundances were noticed in the gut tissue, pharynx and the gut digesta, which shared a striking similarity with the food and culture environments. Additionally, it appears that the microbiota of the sea urchin consisted of a high abundance of Proteobacteria, which is comparable to observations of previously examined marine invertebrate gut microbiota (Van Horn et al., 2011). For example, in the sea slug, members of Alpha-, Beta-, and Gammaproteobacteria have been observed as overrepresented (Devine et al., 2012), and in the gut of the sea cucumber *Apostichopus japonicus*, an echinoderm, it was shown that members of Delta- and Gammaproteobacteria are dominant (Gao et al., 2014).

The luminal surface of the gut contained a low overall bacterial diversity, but a high relative abundance of order Campylobacterales of class Epsilonproteobacteria (Figure 1). It has been reported that representatives from this class have been found to inhabit many ecological niches, both terrestrial and marine, performing a diversity of metabolic functions (Eppinger et al., 2004; Gupta, 2006). In the marine environment, members of Epsilonproteobacteria have been associated as gill symbionts of hydrothermal vent dwellers such as the bivalve *Bathymodiolus azoricus* (On, 2001) and gastropod *Cyathermia naticoides* (Zbinden et al., 2014); as residents of other bivalves such as mussels *Brachidontes* sp. of marine lakes (Cleary et al., 2015) and the Chilean oyster *Tiostrea chilensis* (Romero et al., 2002); as epibionts of crustaceans such as *Kiwa puravida* (Goffredi et al., 2014); and lastly, as gut microbial inhabitants of the aquacultured Norway lobster *Nephrops norvegicus* (Meziti et al., 2012) and hydrothermal vent dwelling shrimp, *Rimicaris exoculata* (Durand et al., 2010). Therefore the commonality of the occurrence of Epsilonproteobacteria in marine invertebrates and the sea urchins in our study may indicate a mutual benefit between the bacterial taxa and the host, perhaps at the physiological and nutritional level.

Further analysis of the lower level of taxonomic groups within Campylobacterales showed 37 oligotypes across all ten samples, with Oligotype 1 displaying a dominant presence in the gut tissue (Figure 2). This suggests that Oligotype 1 is the preferred bacterial group in the sea urchin gut. Additionally, a MEGABLAST search of the representative sequence of the highly abundant gut tissue Oligotype 1 revealed an uncultured species of *Arcobacter* sp., as well as *Sulfuricurvum* sp., and *Arcobacter bivalviorum* (Identities >90%). In a previous study, Epsilonproteobacteria clones identified as *Arcobacter* sp. were found to be associated with marine organisms, including shrimp species (*Rimicaris exoculata*) and the Chilean oyster (*Tiostrea chilensis*) (Romero et al., 2002; Durand et al., 2010). Taxonomic groups similar to Oligotype 1 were also found in the sea urchin feed and water samples, although to a much lesser extent, suggesting that the culture environment may have contributed to the high abundance of Oligotype 1 in the gut tissue microbial ecosystem following proliferation (Figure 2).

As food enters the digestive tract of sea urchins, it is enveloped in a mucosal film that remains intact even after egestion, as a microbial-enriched fecal pellet (Sauchyn et al., 2011; Holland, 2013). The microbiota of the gut digesta and egested fecal pellets both contained a high abundance of Gammaproteobacteria, specifically *Vibrio* of family Vibrionaceae (Figure 1). In as early as 1954, Lasker and Geise reported colonization of bacteria in the gut digesta through microscopic observation (Lasker and Giese, 1954). Similarly in our study, a preliminary examination of the egested fecal pellets using transmission electron microscopy showed comma, round, and rod shaped structures, which appeared to be bacteria resembling *Vibrio, Arcobacter* and *Agarivorans*, genera later determined by NextGen sequencing using the Illumina MiSeq sequencing platform (Supplementary Figure 2). Besides morphological studies, much attention has been allotted to the bacteria colonizing the ingested feed of the sea urchin, with many investigations implicating those bacteria as both crucial to the digestive physiology of the sea urchin, as well as an enriched source of nutrients to organisms at various trophic levels in the hydrosphere (Johannes and Satomi, 1966; Koike et al., 1987; Sauchyn et al., 2011). Previous studies on the gut related microbiota of sea urchins have described the potential symbiotic support of certain strains of *Vibrio* to the sea urchin *Strongylocentrotus droebachiensis*, specifically nitrogenase activity, which is necessary for nitrogen fixing in the assimilation of proteins in sea urchin gonad (Fong and Mann, 1980; Guerinot et al., 1982).

Trends of microbial ecology in the sea urchin have been suggested by Guerinot and Patriquin (1981), who proposed a possibility of an endemic microbiota that will not dissociate from the gut wall of the sea urchin as food transits through the digestive tract (Guerinot and Patriquin, 1981; Lawrence et al., 2013). Evidence of this can be observed in the current study, as the gut digesta and egested fecal pellets were heavily dominated by *Vibrio* species, which were not observed to be significant in the gut tissue (Figure 1). Moreover, a unique oligotype (Oligotype 1) was observed in the gut tissue, which did not appear to be as significant in the gut digesta and egested fecal pellets. This indicates that there is a preference by the host to select specific microbial taxa, perhaps necessary for their nutrition and health (Thorsen, 1998). Moreover, the pharynx tissue shared many of the bacterial taxa of the sea urchin feed (Figure 1), suggesting a likely influence and transmittance of microbes from the food source, which is supported through oligotype analysis (Figure 2), a trend also observed by Meziti et al. (2007) in *P. lividus* (Meziti et al., 2007). The outcome of this study has established for the first time the microbial community composition in the sea urchin *L. variegatus* gut ecosystem, as well as its culture environments, using NextGen sequencing and bioinformatics to achieve taxonomic coverage at the highest level. Future evaluation of the functional metagenomics of the gut microbiome of *L. variegatus* is warranted to establish the role of the microbial community associated with the digestive physiology, nutritional and other health benefits of this animal.

### Conflict of interest statement

The authors declare that the research was conducted in the absence of any commercial or financial relationships that could be construed as a potential conflict of interest.
