# Supplementary material for: An abundance of Epsilonproteobacteria revealed in the gut microbiome of the laboratory cultured sea urchin, Lytechinus variegatus
Source: Front Microbiol. 2015 Oct 13;6:1047. doi: 10.3389/fmicb.2015.01047 (PMC4602345; doi:10.3389/fmicb.2015.01047)
Supplement: Supplementary file 1 [file Supplementary_Table_1.PDF]

Supplementary Table 1. The presence/absence of different taxa (up to genus level) classified from the 10 samples used in this study by using RDP Classifier (at 80% confidence) within the QIIME (v1.7.0).

Taxon	Tank water	Sea Urchin Feed	UR1 Pharynx Tissue	UR2 Pharynx Tissue	UR1 Gut Tissue	UR2 Gut Tissue	UR1 Gut Digesta	UR2 Gut Digesta	UR1 Egested Fecal Pellet	UR2 Egested Fecal Pellet
**Phylum Actinobacteria**										
Actinomyces	-	-	+	+	-	-	-	-	-	-
Aeromicrobium	-	-	+	-	-	-	-	-	-	-
Bifidobacterium	-	+	+	+	-	+	-	-	-	-
Corynebacterium	-	+	+	+	-	-	-	-	-	-
Dietzia	-	-	+	-	-	-	-	-	-	-
Microbispora	-	-	-	+	-	-	-	-	-	-
Micrococcus	-	-	+	+	-	-	-	-	-	-
Rothia	-	-	+	+	-	-	-	-	-	-
**Phylum Bacteroidetes**										
Bacteroides	+	+	+	+	-	+	-	-	-	-
Cloacibacterium	-	+	+	+	-	-	-	-	-	-
Cytophaga	-	-	-	-	-	-	+	+	-	+
Flavobacterium	-	+	+	+	-	-	-	-	-	-
Lewinella	+	-	-	-	-	-	-	-	-	-
Lutimonas	-	-	-	-	-	-	+	-	-	-
Odoribacter	-	-	+	-	-	-	-	-	-	-
Parabacteroides	-	-	+	+	-	-	-	-	-	-
Prevotella	-	+	+	+	-	-	-	-	-	-
Sediminibacterium	-	-	-	+	-	-	-	-	-	-
**Phylum Chloroflexi**										
Ardenscatena	+	-	-	-	-	-	-	-	-	-
**Phylum Firmicutes**										
Allobaculum	+	+	+	+	-	+	-	-	-	-

Bacillus	-	+	+	+	-	-	-	-	-	-
Blautia	-	+	-	+	-	+	-	-	-	-
Carnobacterium	-	-	+	-	-	-	-	-	-	-
Enterococcus	-	+	+	+	-	-	-	-	-	-
Faecalibacterium	-	+	-	+	-	+	-	-	-	-
Lactobacillus	+	+	+	+	-	-	-	-	-	-
Lactococcus	-	+	+	+	-	-	-	-	-	-
Leuconostoc	-	+	-	-	-	-	-	-	-	-
Oscillospira	+	+	+	+	-	-	-	-	-	-
rc4-4	-	-	+	+	-	-	-	-	-	-
Roseburia	-	+	-	-	-	+	-	-	-	-
Ruminococcus	+	+	+	+	-	-	-	-	-	-
Ruminococcus	-	+	+	+	-	-	-	-	-	-
Staphylococcus	-	+	+	+	-	-	-	-	-	-
Streptococcus	-	+	+	+	-	-	-	-	-	-
Turicibacter	-	+	+	+	-	-	-	-	-	-
**Phylum Fusobacteria**										
Propionigenium	+	-	-	+	-	-	-	+	+	+
Psychrilyobacter	-	-	-	-	-	-	-	+	-	+
**Phylum Planctomycetes**										
Planctomyces	+	-	-	-	-	-	+	+	-	+
**Phylum Proteobacteria**										
Acinetobacter	-	+	+	+	-	-	-	-	-	-
Agarivorans	+	-	+	+	-	-	+	+	+	+
Aggregatibacter	+	-	-	-	-	-	-	-	-	-
Agrobacterium	-	+	+	+	-	-	-	-	-	-
Alkanindiges	-	+	+	+	-	-	-	-	-	-
Arcobacter	+	-	+	+	-	+	+	+	+	+
Azorhizobium	-	+	+	+	-	-	-	-	-	-
Azospirillum	-	+	+	+	-	-	-	-	-	-
Bacteriovorax	-	-	-	-	-	-	-	-	-	+

Bdellovibrio	+	-	+	+	-	-	-	-	-	-
Brevundimonas	-	-	+	-	-	-	-	-	-	-
Citrobacter	-	+	+	+	-	-	-	-	-	-
Cobetia	-	+	-	-	-	-	-	-	-	-
Desulfovibrio	+	-	-	-	-	-	-	-	-	+
Devosia	-	+	+	+	-	-	-	-	-	-
Enhydrobacter	-	-	+	+	-	-	-	-	-	-
Francisella	+	-	-	-	-	-	-	-	-	-
HTCC	+	-	-	-	-	-	-	-	-	-
Janthinobacterium	-	+	+	+	-	-	-	-	-	-
Kaistobacter	-	+	+	+	-	-	-	-	-	-
Leucothrix	+	-	-	-	-	-	-	-	-	-
Limnobacter	-	-	+	+	-	-	-	-	-	-
Limnohabitans	-	+	+	+	-	-	-	-	-	-
Lysobacter	-	+	+	+	-	-	-	-	-	-
Marinicella	+	-	-	-	-	-	-	-	-	-
Marinomonas	-	+	-	-	-	-	-	-	-	-
Massilia	-	+	+	+	-	-	-	-	-	-
Methylobacterium	-	+	+	+	-	-	-	-	-	-
Moritella	+	-	-	-	-	-	-	-	+	-
Mycoplana	+	+	+	+	-	+	+	+	-	-
Neisseria	-	-	+	+	-	-	-	-	-	-
Novispirillum	-	+	+	+	-	-	-	-	-	-
Novosphingobium	-	-	+	+	-	-	-	-	-	-
Oceaniserpentilla	+	-	-	-	-	-	-	-	-	-
Octadecabacter	+	-	-	-	-	-	-	-	-	-
Paracoccus	-	+	+	+	-	-	-	-	-	-
Peredibacter	-	+	+	+	-	-	-	-	-	-
Phaeobacter	-	-	-	-	-	-	+	-	-	-
Phenylobacterium	-	+	+	+	-	-	-	-	-	-
Photobacterium	+	+	+	-	-	-	+	+	+	+

Pleomorphomonas	-	+	+	+	-	-	-	-	-	-
Plesiocystis	+	-	-	-	-	-	-	-	-	-
Pseudoalteromonas	+	+	-	-	-	-	-	-	-	-
Pseudomonas	-	+	+	+	-	-	-	-	-	-
Psychromonas	-	+	-	-	-	-	-	+	-	-
Rheinheimera	-	+	+	+	-	-	-	-	-	-
Rhodobacter	-	-	+	+	-	-	-	-	-	-
Rhodospirillum	-	+	+	+	-	-	-	-	-	-
Rubellimicrobium	-	+	+	+	-	-	-	-	-	-
Shewanella	+	+	+	+	-	-	+	+	+	+
Sphingomonas	-	+	+	+	-	-	-	-	-	-
Sphingopyxis	-	-	+	-	-	-	-	-	-	-
Sulfurimonas	-	-	+	+	-	+	+	+	+	+
Thalassomonas	+	-	-	-	-	-	-	-	-	-
Thalassospira	+	-	-	-	-	-	-	-	-	-
Thiothrix	+	-	-	-	-	-	-	-	-	-
Vibrio	+	+	+	+	-	+	+	+	+	+
Vitreoscilla	-	+	-	-	-	-	-	-	-	-
Vogesella	-	+	+	+	-	-	-	-	-	-
**Phylum Tenericutes**										
Candidatus Hepatoplasma	-	-	-	-	-	+	-	-	-	-
