# Supplementary figures and images for: An abundance of Epsilonproteobacteria revealed in the gut microbiome of the laboratory cultured sea urchin, Lytechinus variegatus
Source: Front Microbiol. 2015 Oct 13;6:1047. doi: 10.3389/fmicb.2015.01047 (PMC4602345; doi:10.3389/fmicb.2015.01047)

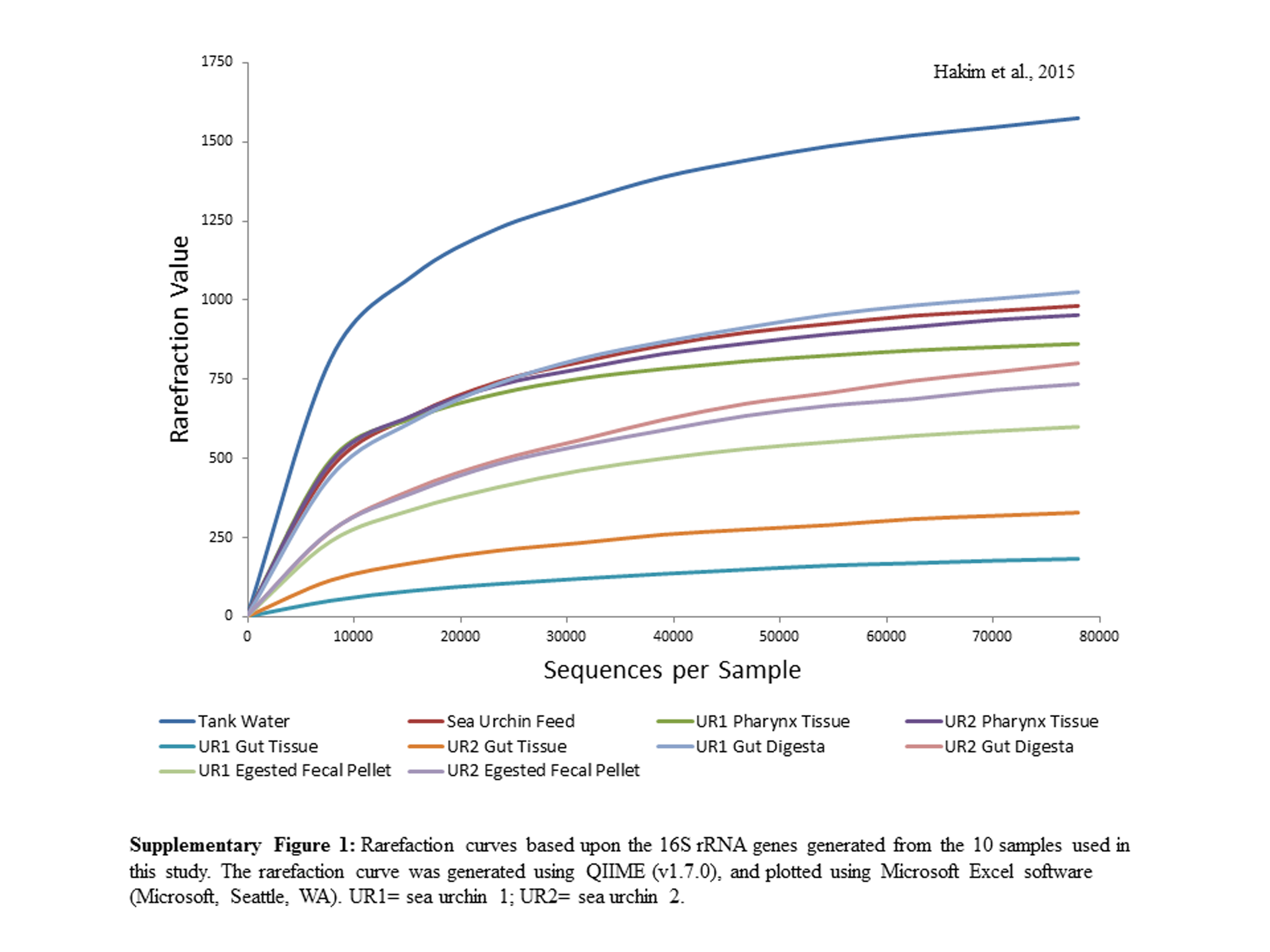

Supplement: Supplementary file 3 [file Supplementary_Figure_1.TIF]

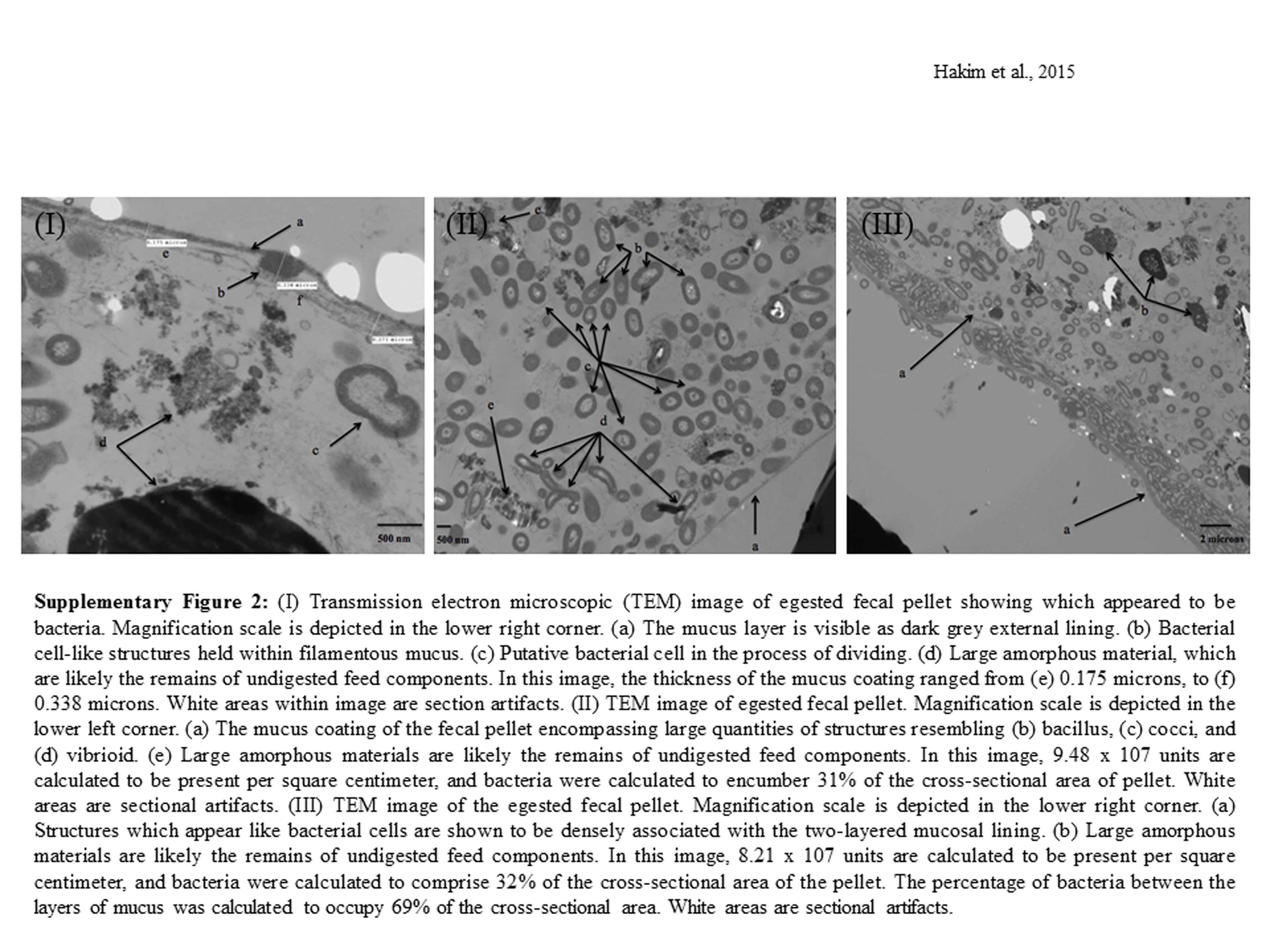

Supplement: Supplementary file 4 [file Supplementary_Figure_2.TIF]
